# Supplementary material for: An evolutionary analysis of supply chain collaborative information sharing based on prospect theory
Source: PLoS One. 2024 Mar 15;19(3):e0298355. doi: 10.1371/journal.pone.0298355 (PMC10942068; doi:10.1371/journal.pone.0298355)
Supplement: S1 File — (DOCX) [file pone.0298355.s001.docx]

My minimal data set are as follows:

The loss aversion coefficient is $\lambda=2.25$, risk coefficients are $\alpha_{1}=\alpha_{2}=\beta_{1}=\beta_{2}=0.88$, amount of information shared by suppliers and retailers are $b_{1}=b_{2}=250$, complementarity coefficients are $t_{1}=t_{2}=0.5$, information sharing costs are $c_{1}=c_{2}=30$, additional benefits are $m=n=100$, initial information sharing probability of the supplier is $x=0.5$ and initial information sharing probability of the retailer is $y=0.5$.
